# COVID-19 in the elderly: A Malaysian perspective

**DOI:** 10.7189/jogh.10.020370

**Published:** 2020-12

**Authors:** Nazri Mustaffa, Si-Yuen Lee, Siti Nurbaya Mohd Nawi, Mohd Jazman Che Rahim, Yong Chuan Chee, Alwi Muhd Besari, Yeong Yeh Lee

**Affiliations:** Department of Medicine, School of Medical Sciences, Universiti Sains Malaysia, Kota Bharu, Malaysia

Coronavirus disease 2019 (COVID-19) was first reported in December 2019 and has since spread globally, currently affecting more than 180 countries, infected more than 3 million individuals, and caused almost 300 000 deaths. Malaysia, a multi-cultural country with a population of approximately 30 million, is among the most affected countries in Southeast Asia. As of 26 May 2020, publicly available information indicated that the local COVID-19 mortality rate stood at 1.5% with 115 reported deaths among 7604 infected persons. **Table 1** summarizes publicly accessible data from the Department of Statistics, Malaysia regarding the number of COVID-19 deaths in Malaysia; higher figures tend to be seen in states that have a larger elderly population.

**Table 1:** Malaysian COVID-19 statistics, according to state (as of 26 May 2020) [1]

State	Confirmed cases, n	Deaths, n	Population (in thousands), n	Population 65+ (in thousands), n
Selangor	1838	22	6715.6	401.3
Johor	671	19	3926.5	292.2
Perak	255	6	2611.6	274.9
Sarawak	549	17	2907.5	227.1
Kedah	95	1	2267.5	191.6
Penang	121	1	1806.5	184.6
Sabah	316	4	4047.0	172.8
FT Kuala Lumpur	1378	17	1910.7	137.9
Pahang	344	7	1750.1	131.7
Kelantan	156	3	1959.7	127.7
Negeri Sembilan	793	8	1162.6	95.8
Melaka	216	5	960.5	92.2
Terengganu	111	1	1294.1	83.4
Perlis	18	2	264.7	23.1
FT Labuan	16	0	103.1	4.8
FT Putrajaya	93	1	94.6	1.7

FT Kuala Lumpur – Federal Territory of Kuala Lumpur, FT Labuan – Federal Territory of Labuan, FT Putrajaya – Federal Territory of Putrajaya (Federal Territory of Kuala Lumpur is located within the state of Selangor)

*Source: Publicly accessible data provided by the Department of Statistics, Malaysia [1].

Although the overall fatality of COVID-19 is low, older adults and patients with comorbidities are more likely to have a more severe course of disease and higher risk of mortality. Statistics from the Ministry of Health, Malaysia indicate that a large majority of patients who succumbed to COVID-19 had one or more concomitant chronic diseases. (unpublished data) Thus, the elderly are particularly vulnerable to COVID-19 due to the complexities of their medical problems which are further compounded by advanced frailty; a United Nations policy brief on the impact of COVID-19 on older persons was recently released to assist in identifying and addressing these concerns [2].

The challenges in health care delivery and accessibility for this at-risk population may be multifactorial; sociocultural (including spiritual well-being) and environmental factors (eg, long-term care facilities) are likely important contributing influences and may be uniquely different in Malaysia as compared to other countries. In addition, many may have cognitive impairment, multiple health conditions, physical (and emotional) dependency with underlying nutritional deficiencies which could further complicate the situation. Finally, the increasingly important role of telemedicine use among the elderly is another issue that warrants attention.

## CHALLENGES IN HEALTH CARE DELIVERY AND ACCESSIBILITY

Even before the emergence of COVID-19, older persons have already faced significant barriers in accessing health services and support such as transportation difficulties, higher medical costs, fear of discovering serious illness and lack of doctors’ responsiveness to concerns [3]. Nevertheless, global reports indicate a precipitous drop in the number of patients seeking non-COVID care, suggesting that some patients would rather avoid hospital visits by staying at home instead of risking potential coronavirus exposure [4]. This phenomenon may also be confounded by the various *cordon sanitaire* orders that have been declared by governments, which aim to contain the spread of the disease by limiting public movement. In Malaysia, the application of the Movement Control Order (MCO) has been used to keep residents indoors, leading to considerable concern regarding diagnosis and treatment delays that could adversely impact patient outcomes. These movement restrictions as well as limited transportation options may further exacerbate ongoing health care delivery and accessibility issues.

## CHALLENGES IN LONG-TERM CARE SETTINGS

Residents in long term care are more susceptible to severe illness with COVID-19 owing to their generally frailer condition and the burden of chronic diseases [5]. Currently there has been no report of local COVID-19 outbreaks in Malaysian long-term care facilities; however, given that the majority of them are concentrated in larger cities where active cases of COVID-19 are detected, significant concern of outbreaks is still warranted.

The model of long-term care in Malaysia may be different as compared to developed countries. Some facilities are managed by the government whilst others are privately run. Most offer dormitory-type sleeping arrangements ranging from 2-6 beds per dormitory; many are largely supported by donations. Well-known issues such as a shortage of adequately trained staff, limited funding and resources raise concerns regarding the ability to contain an outbreak if this were to happen. In response, the Malaysian Society of Geriatric Medicine has published interim recommendations regarding the COVID-19 pandemic that provide guidelines to reduce risk of outbreaks eg, by suspending group activities, restricting visitors, practicing frequent hand hygiene, implementing temperature checks on staff, and designating isolation areas for suspected COVID-19 cases. Guidance is also provided with regards to in- and out-bound patient transfers at these facilities [6].

## PSYCHOLOGICAL AND SPIRITUAL WELL-BEING

Subjective well-being (SWB) is a parameter used to assess the psychological well-being, life satisfaction, global happiness, and quality of life of a target population. Previous experiences in countries such as Hong Kong which had faced both the 2003 Severe Acute Respiratory Syndrome (SARS) and the 2009 influenza A virus subtype H1N1 epidemics found that the majority of older persons there maintained a stable overall SWB despite the outbreaks. This was attributed to successful targeted community outreach programs that assisted with coping, a high psychological resilience coupled with an existing strong culture of neighborhood bonding and communal living among the elderly. However, lower SWB was seen amongst the elderly who lived in high-incidence outbreak districts, specifically among those of a female gender, with a lower education level or being unemployed [7]. In Malaysia, factors that are associated with poor SWB scores among the elderly are an increasing age, being of female gender or unmarried, having morbidities or being in poverty [8].

In Malaysia many of the elderly persons would have been admitted to nursing homes after being abandoned at hospitals, whilst some were often brought in after being found on the streets. These elderly residents would be likely to have poor baseline SWB due to the multiple factors that were mentioned earlier such as poor health, advanced age and being in poverty. Although the overall effect of the pandemic on the SWB of Malaysian nursing home residents is yet to be known, it is still important for us to consider its implications as this could heavily impact the well-being of this vulnerable population.

## NUTRITIONAL CHALLENGES

In Malaysia, limited personal mobility following the application of social distancing orders may lead to a new set of problems. With an advancing age there is also the risk of having one or more co-morbidities; prior to the COVID-19 pandemic the presence of multiple chronic diseases were already recognised as being associated with an increased risk as well as prevalence of malnutrition that could lead to more severe consequences [9]. Thus, there is a need to ensure that this group of patients receive adequate nutritional support whilst being confined to their residences.

The European Society for Clinical Nutrition and Metabolism (ESPEN) for example, has provided an official statement and practical guidance regarding this matter [10]. Essentially, the statement highlights the importance of recognising and treating nutritional deficits of those with COVID-19, an issue which is imperative when considered within the context of treating elderly patients with multiple co-morbidities.

**Figure:**
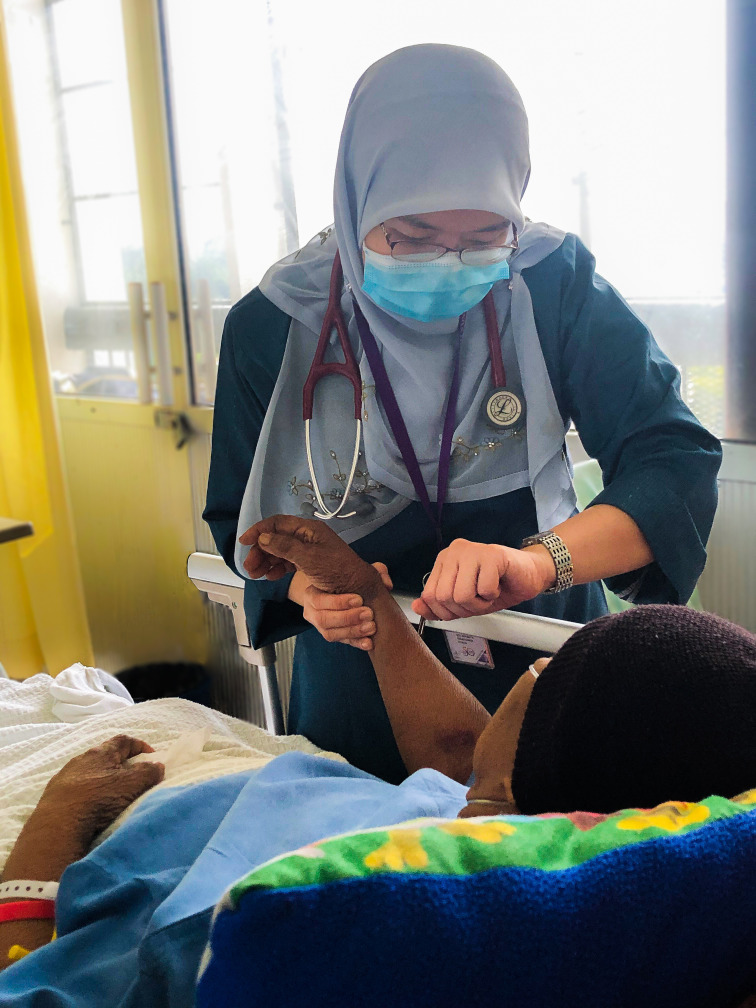
Photo: From the collection of Siti Nurbaya Mohd Nawi, used with permission.

## MOVING FORWARD: CHALLENGES IN ADOPTION OF TELEMEDICINE SERVICES

During the COVID-19 outbreak, telemedicine has emerged as an important tool for the management of communicable and non-communicable diseases among the elderly. Audio and/or virtual platforms could be set up using telephones, smartphones and webcam-enabled computers, allowing physicians to provide consultation services that address the home health care needs of older patients, as well as providing effective pre-hospital screening for early symptoms and signs of COVID-19.

Nonetheless, implementing telemedicine services among the elderly is not without its challenges; most countries lack a regulatory framework to authorize, integrate and reimburse telemedicine services in outbreak and emergency situations [11]. Although telemedicine is an integral part of Malaysian health care planning it has not however, been fully implemented within the public health service. As it is, several state general hospitals and designated hospitals for COVID-19 have started to offer public telemedicine services. For example, the Geriatric Unit of Sungai Buloh Hospital, an infectious disease centre for treating COVID-19 has provided virtual clinics during this pandemic.

Several other issues may also negatively impact the use of telemedicine among older patients. Some consultations require physical examination that cannot be done remotely, whilst others may need further diagnostic assessments. It is important that health care providers highlight the limitations of telemedicine and inform their clients of alternative methods. Nevertheless, it is believed that the rapid scale-up of telemedicine use during the COVID-19 pandemic will have long-term implications for access to primary, specialty and subspecialty care among older persons. With an increasing number of health providers and practices being equipped with the infrastructure needed to provide telemedicine services, this will likely continue to be offered on a routine basis beyond the pandemic.

## CONCLUSION

There are numerous challenges which are being faced by the elderly in Malaysia. Even though the issues that have been highlighted may not be completely unique to the local population, the current COVID-19 pandemic has exacerbated previous concerns regarding this particularly vulnerable group of society. Appropriate health delivery within the context of social distancing, maintaining well-being whilst in long-term health care facilities, psychological and spiritual welfare, and nutritional matters are all important points that are applicable globally as well as within a more local Malaysian context. Finally, planning for the future there is a need to leverage further on telehealth/telemedicine services in order to optimise health delivery and availability.
